# Pressure-dependent NOS activation contributes to endothelial hyperpermeability in a model of acute heart failure

**DOI:** 10.1042/BSR20181239

**Published:** 2018-11-23

**Authors:** Andreia Z. Chignalia, Ayman Isbatan, Milan Patel, Richard Ripper, Jordan Sharlin, Joelle Shosfy, Barry A. Borlaug, Randal O. Dull

**Affiliations:** 1 Department of Anesthesiology, University of Illinois at Chicago, Chicago, IL 60612, U.S.A.; 2 Research and Development Service, Jesse Brown Veterans Affairs Medical Center, 820 S Damen Ave., Chicago, IL 60612, U.S.A.; 3 Department of Cardiovascular Medicine, Mayo Clinic and Foundation, 200 First St SW, Rochester, MN 55905, U.S.A.; 4 Department of Anesthesiology, University of Arizona College of Medicine and Banner-University Medical Center, Tucson, AZ 85724, U.S.A.

**Keywords:** heart failure, mechanotransduction, nitric oxide synthase, oxidative stress, pulmonary edema

## Abstract

**Aims:** Acute increases in left ventricular end diastolic pressure (LVEDP) can induce pulmonary edema (PE). The mechanism(s) for this rapid onset edema may involve more than just increased fluid filtration. Lung endothelial cell permeability is regulated by pressure-dependent activation of nitric oxide synthase (NOS). Herein, we demonstrate that pressure-dependent NOS activation contributes to vascular failure and PE in a model of acute heart failure (AHF) caused by hypertension.

**Methods and results:** Male Sprague–Dawley rats were anesthetized and mechanically ventilated. Acute hypertension was induced by norepinephrine (NE) infusion and resulted in an increase in LVEDP and pulmonary artery pressure (P_pa_) that were associated with a rapid fall in P_a_O_2_, and increases in lung wet/dry ratio and injury scores. Heart failure (HF) lungs showed increased nitrotyrosine content and ROS levels. L-NAME pretreatment mitigated the development of PE and reduced lung ROS concentrations to sham levels. Apocynin (Apo) pretreatment inhibited PE. Addition of tetrahydrobiopterin (BH4) to AHF rats lung lysates and pretreatment of AHF rats with folic acid (FA) prevented ROS production indicating endothelial NOS (eNOS) uncoupling.

**Conclusion:** Pressure-dependent NOS activation leads to acute endothelial hyperpermeability and rapid PE by an increase in NO and ROS in a model of AHF. Acute increases in pulmonary vascular pressure, without NOS activation, was insufficient to cause significant PE. These results suggest a clinically relevant role of endothelial mechanotransduction in the pathogenesis of AHF and further highlights the concept of active barrier failure in AHF. Therapies targetting the prevention or reversal of endothelial hyperpermeability may be a novel therapeutic strategy in AHF.

## Introduction

Acute increases in left ventricular end diastolic pressure (LVEDP) and pulmonary capillary pressure (P_pc_) can cause rapid and severe pulmonary edema (PE) [1]. Clinically, however, the severity of PE is often out of proportion to the increase in P_pc_ suggesting that other mechanisms may be operational. To understand this clinical disparity, we explored the relationship between sudden changes in hydrostatic pressure and acute changes in endothelial permeability. We have previously demonstrated that small changes in hydrostatic pressure produced large, rapid, and sustained increases in endothelial permeability, an effect mediated by nitric oxide (NO) [2] and reactive oxygen species (ROS) [3]. In fact, NO-mediated increases in endothelial permeability during increased hydrostatic pressure are common throughout the vascular system [4,5]. Thus, increases in both pressure and shear stress can activate nitric oxide synthase (NOS) and increase endothelial NO. Nitrosylation of adherens junction proteins is a trigger for junctional disassembly and results in acute increases in endothelial permeability [6]. The mechanisms whereby ROS contributes to endothelial hyperpermeability, however, are less understood. Reactive oxygen species are generated in a controlled and compartmentalized manner in the vasculature and although NADPH oxidases (Nox) are considered to be the main source of ROS in the vascular system, there is no evidence that Nox regulates barrier function; thus, other sources for ROS production, such as uncoupled endothelial NOS (eNOS) [7], may play a role in endothelial hyperpermeability and PE.

Interestingly, the majority of patients with chronic heart failure (HF) do not develop frank PE, even when P_pc_ is elevated [8,9] suggesting that alterations in endothelial signaling pathways, structural alteration of the vascular wall, or both, contribute to compensatory mechanism(s) that protect against sustained high pressure. We recognize that while NO causes acute changes in endothelial permeability, organic nitrites are used to treat pulmonary congestion and mitigate the symptoms of chronic HF. This paradox suggests, perhaps, that the physiological response of the pulmonary vasculature to NO and/or oxidative stress changes during the progression from acute HF (AHF) to chronic HF and, therefore, warrants further mechanistic characterization. Understanding the early mechanisms that regulate disease progression maybe an important step to better understand this paradox and improve therapeutic approaches to HF.

Herein, we describe a series of studies demonstrating the contribution of NOS activation to acute endothelial barrier failure in a model of AHF. The results demonstrate that eNOS has a dual role in AHF-related PE: it acts by increasing NO concentrations and second as a source of superoxide anion production leading to endothelial hyperpermeability and pulmonary vascular failure. Inhibition of NOS attenuates the physiological and histological pulmonary changes associated with AHF. The novel findings demonstrate that pressure-dependent NOS-activation results in enhancement of endothelial permeability and rapid PE. This represents a major paradigm shift in our understanding of hydrostatic PE and may lead to new therapeutic strategies to re-establish barrier integrity.

## Methods

### Reagents

NADPH, Norepinephrine (NE), Dihydroethidium (DHE), protease inhibitor cocktail, and Krebs Ringer Buffer were bought from from Sigma Co, Ltd (St. Louis, MO); BSA was purchased from Proliant Biologicals (Boone, IA); antibodies were bought from EDM Millipore (Billerica, MA), Aviva Systems Biology (San Diego, CA) and Cell Signaling (Danvers, MA); Lucigenin and L-NAME were bought from Cayman Biochemicals; apocynin was bought from Calbiochem.

### Animals

Animal studies were approved by the University of Illinois Institutional Animal Care and Use Committee. The investigation conforms to the *Guide for the Care and Use of Laboratory Animals* published by the U.S. National Institutes of Health. Male Sprague–Dawley rats were divided in the following groups: Sham, AHF; L-NAME + AHF (L-NAME + AHF); L-NAME; Apocynin (Apo); Apocynin + AHF (Apo + AHF); Ethanol (EtOH); Folic Acid (FA) and FA + AHF (FA + AHF).

### AHF model

Rats received a NE infusion starting at 7 μg/Kg/min and titrated to a mean arterial pressure (MAP) of 150 mm Hg for 2 h. Sham rats received a lactated ringers infusion (1.5 ml/h). For specific experiments, L-NAME or Apo (200 µmol/l/kg) was administered as a bolus over a 10-min period immediately before the induction of AHF. Additional control groups included solo administration of L-NAME and Apo to determine if it had any effects on the measured parameters independent of NE infusion. In order to assess the role of mechanotransduction in the development of PE during AHF, a non-hypertensive dose of L-NAME was chosen to be used in this model. With this approach, although increased mechanical forces and hemodynamics of HF are still present, we can specifically assess the contribution of NOS signaling to PE development.

### Arterial blood gas analysis

Arterial blood gases, hematocrit (HCT) and pH were measured in 250 µl of whole blood using a GEM Premier 3000 machine (Instrumentation Laboratory, Orangeburg, NY), according to manufacturer’s instructions.

### Hemodynamics

Left ventricular end systolic pressure (LVESP), LVEDP, and pulmonary artery (PA) pressures were measured using a saline-filled PE tubing (PE 50) connected to an arterial pressure transducer interfaced to a TAM-A amplifier (Harvard Apparatus, Holliston, MA).

### Lung wet-to-dry ratio

Wet-to-dry (W/D) ratios were determined after drying lungs for 24 h at 60°C.

### Lung injury score

Lung injury was assessed by five blinded investigators and scored based on perivascular cuffing (PVC) and intra-alveolar hemorrhage (IAH). Total lung injury score (LIS) was determined by the weighted average of PVC and IAH.

### Isolated perfused lung preparation

The rat isolated perfused lung preparation was used as previously described [2,10]. Briefly, rats were anesthetized and mechanically ventilated. The PA and left atria were cannulated and lungs were perfused with Krebs–Ringer bicarbonate solution containing 3% BSA. Lungs were exposed to low pressure (6 cm H_2_O) or high pressure (12 cm H_2_O) for 60 min.

To assess if NE could induce lung hyperpermeability, 10^−6^ mol/L of NE was added to the perfusate reservoir and circulated for 1 h at low pressure. Lung W/D ratios were determined.

To assess if NE had a direct effect on lung vasculature, a concentration–response curve for NE (10^−8^ to 10^−3^ mol/L) was performed and pulmonary artery pressure (P_pa_) was recorded.

### BAL albumin content

BAL albumin content was assessed using a fluorimetric detection kit (Active Motif, Carlsbad, CA) according to manufacturer’s instructions.

### MPO activity

MPO activity was measured in lung tissue and in BAL as previously described [11].

### Immunoblotting

#### NOS, Nox, and NO production

Total eNOS, iNOS, Nox1, and Nox2 expression was assessed in whole lung lysates. eNOS activity was determined by p-Ser^1177^/eNOS ratio; NO production was assessed for nitrotyrosine content as a marker for mechanotransduction activation. Immunoblots were performed as previously described [12]. Signal was detected by chemiluminescence using Li-COR system and band intensities were measured using Image Studio software (Li-COR, Lincoln, NE).

#### eNOS uncoupling

To assess whether eNOS is uncoupled, we performed a low-temperature gradient gel following standard Western blot techniques as previously described [13]. Signal was detected by chemiluminescence using Li-COR system. Band intensities were measured using Image Studio software (Li-COR).

### ROS measurements

#### Lucigenin Enhanced Chemiluminescence

ROS production was assessed by lucigenin enhanced chemiluminescence (ECL) as previously described [12]. To determine NOS-dependent superoxide generation, rats were treated with L-NAME and lucigenin ECL assay was performed. To confirm uncoupled eNOS as a source of ROS production in lung homogenates, we assessed ROS levels in lung lysates with exogenous tetrahydrobiopterin (BH4) supplementation (*in vitro*). Addition of PEG-SOD *in vitro* to lung samples was done as an additional control to show superoxide anion is being detected in this assay.

#### Nox activity

Nox activity was assessed in lung membrane fractions as previously described [12]. Results were normalized by protein content in the samples.

### Statistical analysis

Data are presented as mean ± S.D. Groups were compared using one-way ANOVA or Student’s *t* test as appropriate. Tukey’s post-test was used to compensate for multiple test procedures. *P*<0.05 was considered statistically significant.

## Results

### AHF model

During general anesthesia, baseline MAP was 109.8 ± 10 mm Hg and heart rate (HR) averaged 348/min (Table 1). All hemodynamic variables were stable over the 2-h time period. Acute hypertension was induced by a NE infusion resulting in a rapid increase in MAP to 150 mm Hg and a significant increase in LVESP and LVEDP without changes in HR (see Table 1).

**Table 1:** AHF model

	SHAM	AHF	L-NAME + AHF	L-NAME
**MAP values (mm Hg)**				
Baseline	109.8 ± 10.1	123.8 ± 19.3	117.2 ± 14.3	110.6 ± 15.0
Final (120 min)	105.7 ± 9.7	152.2 ± 22.2^*,‡^	149.9 ± 20.2^*,§,‡^	118.3 ± 25.4
**P_pa_ (mm Hg)**				
Baseline SBP	29.3 ± 3.2	26.6 ± 7.8	27.0 ± 2.6	27.0 ± 2.2
Baseline DBP	4.7 ± 1.1	10.1 ± 5.3	9.3 ± 5.5	11.3 ± 5.9
Final SBP (120 min)	30.3 ± 3.21	39.3 ± 5.9	38.3 ± 8.8	27.3 ± 2.87
Final DBP (120 min)	5.0 ± 1.7	18 ± 6.1^‡^	14.2 ± 7.5^‡^	13.0 ± 7.5
mPAP	13.4 ± 1.0	25.1 ± 6.3*	22.23 ± 7.5	17.8 ± 5.6
**Cardiac parameters**				
HR (beats/min)	348.0 ± 8.2	361.2 ± 16.2	352.3 ± 12.4	344.0 ± 28.6
Baseline LVESP/LVEDP (mm Hg)	138.3 ± 7.6/6.3 ± 0.6	148.6 ± 21.9/9.67 ± 5.6	149.4 ± 19.0/10.4 ± 8.4	152.4 ± 16.62/7.8 ± 3.1
Final (120 min) LVESP/LVEDP (mm Hg)	138.0 ± 0.5/6.0 ± 0.1	229.6 ± 28.1/16.3 ± 9.5^‡^	212.0 ± 35.1/15.2 ± 13.9^‡^	165.6 ± 25.4/10.6 ± 5.7
**Arterial blood gases**				
pH	7.48 ± 0.1	7.159 ± 0.2*	7.50 ± 0.3^†^	7.49 ± 0.1
PaO_2_	109.6 ± 28.5	71.29 ± 11.6*	93.0 ± 18.8	91.8 ± 10.6
PaCO_2_	34.78 ± 2.7	36.50 ± 8.9	39.80 ± 5.7^§^	33.63 ± 10.1
HCT	33.63 ± 4.4	41.20 ± 4.9*	36 ± 5.7	38.57 ± 3.4

Experimental groups: Sham; AHF; L-NAME + AHF (L-NAME bolus followed by NE infusion), and L-NAME (l-NAME bolus followed by lactated ringers infusion); *n*≥3/group. Abbreviations: mPAP, mean P_pa_; PaCO_2_, arterial CO_2_ pressure; PaO_2_, arterial O_2_ pressure.

*P<0.05 compared with Sham. ^†^*P*<0.05 compared with NE. ^‡^*P*<0.05 compared with baseline from same group. ^§^*P*<0.05 compared with l-NAME.

Acute hypertension caused a significant increase in pulmonary artery systolic pressure (PASP, 26 ± 8 compared with 39 ± 16 mm Hg) and a doubling of mean P_pa_ (mPAP; Sham = 13.14 ± 1.0 compared with AHF = 25.1 ± 6.3 mm Hg; Table 1). To characterize the direct effects NE on the pulmonary pressures, we used the *in situ* isolated perfused rat lung preparation and tested if NE infusion (10^−8^ to 10^−3^ mol/L) altered P_pa_. NE had no direct effect on pulmonary vascular pressure (Supplementary Figure S1A), indicating that NE-induced pulmonary hypertension requires an intact circulation.

To clarify if NE *per se* could induce lung hyperpermeability, we used the isolated perfused lung preparation to test the effects of high pressure and NE on lung edema. While high pressure induced lung edema when compared with control group, NE did not alter lung W/D ratio when compared with control group (Supplementary Figure S1B). These results demonstrate that pressure, and not NE, is the main stimulus of endothelial hyperpermeability during AHF.

**Figure 1:**
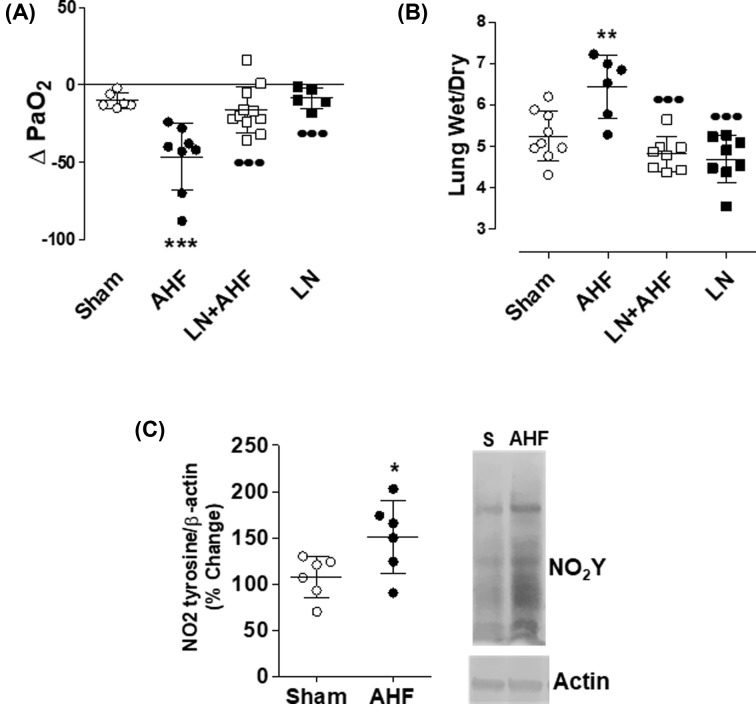
AHF model Rats with AHF showed increased (**A**) ΔPaO_2_; (**B**) Lung W/D ratio; (**C**) Nitrotyrosine (NO_2_ tyrosine) content when compared with sham rats**.** L-NAME administration prior to induction of AHF (LN + AHF) prevented all indices of PE (PaO_2_, lung W/D). L-NAME (LN) bolus had no effect on the measured parameters. *n*≥6/group; *P<0.05 compared with Sham, **P<0.01 compared with Sham, ***P<0.01 compared with Sham, ^●●●^*P*<0.001 compared with AHF.

Elevations in LVEDP and PAP during AHF were associated with the development of PE. P_a_O_2_ dropped on an average to 50% (Figure 1A) and lung W/D ratio increased significantly (5.3 ± 0.52 compared with 6.44 ± 0.75; Figure 1B). Histological evidence of lung injury was manifested as significantly increased PVC and increased IAH. Higher LIS were observed in the dependent lung regions (dorsal) compared with non-dependent regions (ventral). In the dorsal regions, total LIS for Sham were 0.17 ± 0.05 and for AHF rats = 0.38 ± 0.05 (Supplementary Figures S2 and S3).

### Pressure-dependent hyperpermeability during AHF is not an inflammatory process

To determine if AHF and associated increase in P_cp_ caused an increase in leukocytes recruitment to the lungs that contributed to barrier failure, CD45 expression in lung sections, MPO activity in BAL and lung tissue were measured. As shown in Supplementary Figure S4A,B, lung MPO activity was not increased in the AHF BAL and lungs. In the same manner, no differences in CD45 staining was observed in lung sections from AHF lungs when compared with Sham lungs (Supplementary Figure S4D), ruling out increased white blood cell accumulation or activation as a cause for hyperpermeability. We also assessed the levels of interleukin 1-β (IL1-β), macrophage inflammatory protein 2 (MIP2), and tumor necrosis factor-α (TNF-α) by real-time PCR in lung lysates from sham and AHF rats. No differences in IL1-β, TNF-α, and MIP2 were found corroborating our previous data that this is not an inflammatory model of PE during AHF (Supplementary Figure S5).

### Epithelial barrier is not damaged during AHF

To determine if epithelial barrier disruption contributed to the reduction in P_a_O_2_ and increase in lung W/D, albumin content of BAL fluid was measured. Acute increases in pulmonary vascular pressures were not associated with an increase in BAL albumin concentration (Supplementary Figure S4C) when compared with Sham BAL, indicating that the alveolar epithelial barrier remained intact.

### NO in AHF

To determine if acute vascular pressure increased lung NO production, we indirectly assessed NO generation by quantitating nitration of tyrosine residues in lung lysates. As shown in Figure 1C, acute hypertension and associated increase in mPAP resulted in a 40% increase in nitro-tyrosine content, consistent with our previous findings [2] and indicates activation of endothelial mechanotransduction.

### Inhibition of NOS prevents HF-dependent PE

To determine if pressure-dependent PE was caused by NO-mediated barrier failure, rats were treated with the NOS inhibitor, l-NAME, prior to the induction of AHF. l-NAME attenuated PE, returning P_a_O_2_ to Sham values (Figure 1A). In addition, l-NAME abolished the AHF-induced increase in lung W/D ratio (l-NAME + AHF wet/dry = 4.8 ± 0.41 compared with AHF = 6.4 ± 0.75) (Figure 1B). Pretreatment with l-NAME reduced histological evidence of lung injury during AHF. Histological LIS in the dorsal region were reduced in l-NAME + AHF to 0.381 ± 0.05, compared with LIS = 0.287 ± 0.06 (in AHF); *P*=0.0098 (Supplementary Figure S3).

### Reactive oxygen species contribute to PE development in pressure-induced AHF

**Figure 2:**
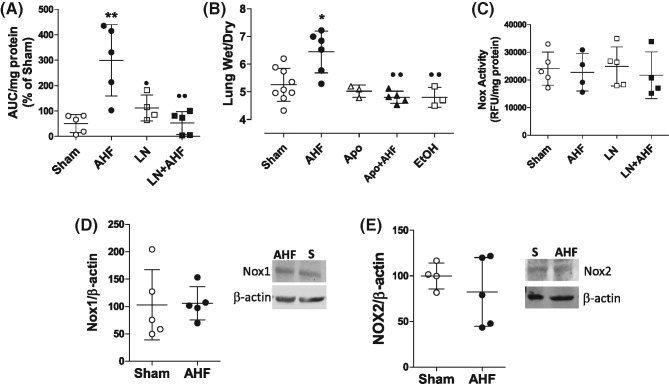
Oxidative stress contributes to PE in AHF Lungs collected from AHF rats showed (**A**) increased ROS levels when compared with lungs collected from sham rats. l-NAME administration before AHF (LN + AHF) prevented increase in lung ROS while L-NAME alone had no effect on ROS production in sham rats. (**B**) Apo attenuated the increase in lung W/D ratio in AHF rats but had no effect on control rat lung W/D. (**C**) Nox activity and (**D**,**E**) expression is not altered in AHF. L-NAME had no effect on Nox activity. *n*≥4/group; *P<0.05 compared with Sham, **P<0.01 compared with Sham, ^●^*P*<0.05 compared with AHF, ^●●^*P*<0.01 compared with AHF.

In order to evaluate if ROS contribute to PE during pressure-induced AHF, we first measured ROS concentrations in lung homogenates from Sham and AHF lungs. AHF lungs showed increased ROS concentration when compared with Sham lungs (299.7 ± 140.3 in AHF compared with 50.91 ± 35.47 in Sham; Figure 2A). The increase in ROS concentration was inhibited when rats were pretreated with L-NAME (L-NAME + AHF: 52.94 ± 45.32) (Figure 2A) suggesting eNOS uncoupling. L-NAME, alone, did not affect ROS concentration (111.7 ± 51.10 in L-NAME compared with 50.91 ± 35.47 in Sham). To investigate if ROS contribute to PE, we assessed lung W/D ratio in rats treated with apocynin prior to induction of AHF. Apo inhibited PE development in AHF (W/D = 4.80 ± 0.21 in Apo + AHF compared with 6.44 ± 0.75 in AHF). Rats that received either apocynin only or EtOH (vehicle used to dissolve Apo) did not show differences in lung W/D ratio (Apo = 5.02 ± 0.7; EtOH = 4.8 ± 0.36) when compared with sham rats (5.25 ± 0.59) (Figure 2B).

### Uncoupled eNOS drives oxidative stress in pressure-dependent AHF

To evaluate the participation of Nox in ROS production during AHF we assessed Nox activity in isolated membrane fraction of lungs collected from Sham and AHF rats. Nox activity was not altered in AHF rats relative to Shams (Figure 2C). To confirm that Nox was not involved in ROS production in this model, we assessed the expression of Nox isoforms 1 and 2 in lung lysates from Sham and AHF rats. No changes were observed in NOX1 and NOX2 content in lung lysates collected from AHF rats when compared with Sham rats (Figure 2D,E).

**Figure 3:**
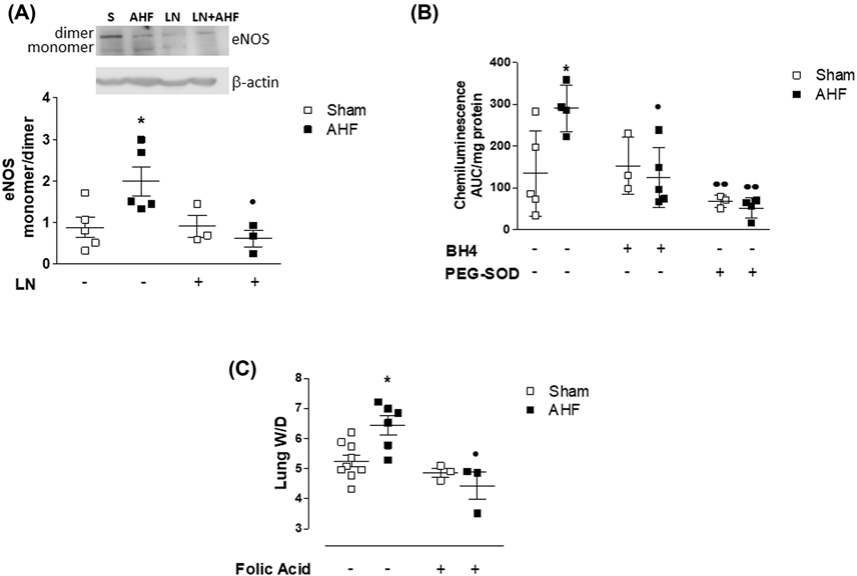
eNOS uncoupling during AHF AHF results in (**A**) eNOS uncoupling, an effect inhibited by pretreatment of hypertensive rats with L-NAME (LN + AHF). l-NAME alone had no effect on eNOS monomer/dimer ratios. (**B**) Addition of BH4 or PEG-SOD to AHF lung lysates decreased ROS. (**C**) Treatment of rats with Folic Acid before induction of AHF resulted in protection from edema development. *P<0.05 compared with Sham, ^●●^p<0.01 compared with AHF. *n*≥3/group.

As L-NAME decreased ROS levels during AHF, we then considered eNOS as a source for oxidative stress in AHF and assessed eNOS uncoupling by Western blot. AHF lungs showed eNOS uncoupling which was not observed in Sham lungs. Pretreatment of AHF rats with L-NAME attenuated eNOS uncoupling during AHF (Figure 3A) thus supporting the findings that L-NAME inhibited ROS production. Collectively, these results suggest that eNOS uncoupling occurs due to excessive eNOS stimulation and the lack of enzyme substrate.

### Mechanism for eNOS uncoupling in AHF

We evaluated the hypothesis that depletion of BH4 was contributing to eNOS uncoupling. Exogenous BH4 was added to lung lysates *in vitro* and ROS production was assessed by lucigenin ECL. As shown in Figure 3B, supplementation of BH4 in lung lysates decreased ROS levels in AHF lungs (from 290.5 ± 55.41 in AHF to 125 ± 71.17 in AHF + BH4). The same effect was observed when PEG-SOD was added to the lysates (51.88 ± 24.13 in AHF + PEG SOD), indicating that superoxide anion was the ROS being measured. These results support the hypothesis that uncoupled eNOS was a source of ROS. To further investigate if uncoupled eNOS contributes to PE development *in vivo*, we treated rats with FA, known to recouple eNOS by promoting BH4 recycling, before inducing AHF and assessed lung W/D ratio. In the presence of FA (FA + AHF group), rats that underwent AHF did not develop PE as evidenced by similar lung W/D ratio when compared with sham rats (4.43 ± 0.78 in FA + AHF compared with 5.2 ± 0.59 in Sham), Figure 3C.

**Figure 4:**
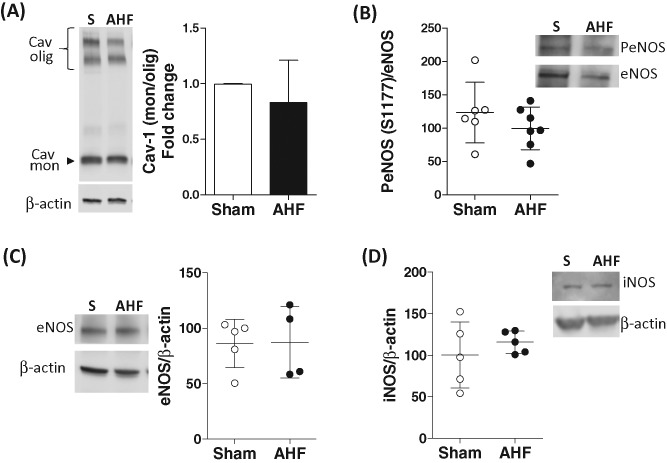
Caveolae and NOS isoforms during AHF AHF did not result in (**A**) caveolae degradation, (**B**) changes in eNOS activity, (**C**) eNOS expression, or (**D**) iNOS content. *n*≥4/group.

We then investigated if caveolae degradation was part of the mechanism(s) leading to eNOS uncoupling in AHF lungs. Western blot analysis of lung homogenates did not demonstrate caveolae degradation (Figure 4A), ruling out caveola deterioration as a pathway to eNOS uncoupling in pressure-dependent AHF. Additionally, we investigated eNOS activity in lung lysates from sham and AHF rats at the final time point of the experimental model (2 h). No significant changes in eNOS activity were observed in AHF lungs when compared with sham lungs (Figure 4B).

### NOS isoforms

In order to determine if differences in NOS isoforms expression occurred during AHF, we assessed eNOS and iNOS expression in lung lysates. No differences in NOS isoform expression was seen between groups (Figures 4C,D), thus ruling out iNOS contribution to increase NO production during AHF.

## Discussion

We used a hemodynamic model of HF that was induced by acute afterload mismatch, resulting in increased LVEDP, increased P_pa_ and subsequently, rapid PE [14–16]. This clinically relevant model allowed us to investigate the role of acute pressure-dependent endothelial mechanotransduction on vascular barrier failure as a mechanism of AHF-associated PE. Acute hypertension and the associated afterload mismatch resulted in: (i) a significant decrease in P_a_O_2_ that occurred within 30 min, (ii) an increase in lung W/D ratio and, (iii) an increase in histological LIS. Inhibition of NOS during AHF prevented the reduction in P_a_O_2_, maintained normal lung W/D ratio, and reduced histological LIS. The major novel finding of the present study is that in the absence of NOS-dependent enhancement in endothelial permeability, acute increases in pulmonary vascular pressure alone are insufficient to cause significant PE. This represents a major paradigm shift in our understanding the pathophysiology of hydrostatic PE.

These findings extend previous work on pressure-dependent endothelial mechanotransduction to a clinically relevant model of AHF. Kuebler et al. (2003) [17] were the first to demonstrate that acute increases in P_pc_ resulted in endothelial NO production. Previous work from our laboratory showed that pressure-dependent NOS activation, increased whole lung filtration coefficient (*K*_f_) and endothelial hydraulic conductivity (*L*_p_) [2,18].

### Pathophysiology of hypertensive AHF

The increase in pulmonary vascular pressure during NE-infusion resulted from a combination of increased LV afterload and a translocation of blood volume from the peripheral circulation to the lungs [19]. The combined effect of increased venous return and increased resistance to pulmonary outflow (increase in LVEDP) resulted in a significant increase in P_pc_ [20]. Because this is a model that combines two vasoactive effectors (pressure and NE) we also sought to identify the primary stimulus for endothelial hyperpermeability. In order to do so, we assessed the direct effects of NE on the pulmonary hemodynamics and edema development in the isolated perfused lung preparation. No changes in P_pa_ or in lung W/D ratio were found, corroborating previous findings by Krishnamoorthy et al. [21]. Given that HR did not increase during NE infusion, the increase in hydrostatic pressure appears the major stimulus for activation of NOS. Collectively, these results indicate that pressure, and not NE, is the main trigger for endothelial hyperpermeability

### Pressure-dependent AHF is not an inflammatory process

Inflammation is a controversial process in HF progression [22]. Although many animal studies suggest that inflammation is a definitive component of AHF, clinical evidence indicates that is not always the case [23]. Particularly, in conditions of elevated pulmonary vascular pressure, localized inflammation may arise from increased leukocyte margination in the lungs [24,25]. In order to evaluate if leukocyte margination contributed to lung endothelial hyperpermeability in the current model of AHF, we assessed myeloperoxidase activation. Myeloperoxidase activity remained unaltered in AHF lungs and BAL. In the context of inflammation, iNOS could be contributing to hyperpermeability in AHF as it has been previously reported to participate in barrier dysfunction [26]. We validate that iNOS expression was not increased in AHF lungs relative to Sham ruling iNOS as a mediator in this model of AHF. Finally, immunohistochemistry for leukocytes failed to demonstrate an increase in immune cells in the lungs from AHF rats and measurement of IL1-β, TNF-α, and MIP2 indicated that production of these cytokines are not altered during AHF.

### L-NAME protects against capillary stress failure

The protective effects of L-NAME on reducing histological indices of lung injury during acutely increased vascular pressure were most notable for the reduction in IAH. This was a novel and an unexpected finding and contributes to the hypothesis that capillary stress failure may be a regulated process [27]. In the dorsal sections of the lung, where hydrostatic pressure would be highest in a supine rat, L-NAME attenuated hypertension-induced IAH. Collectively, these results suggest that pressure-dependent increases in endothelial permeability, including capillary stress failure, occur as a part of NO-mediated process. Studies are underway to further characterize the role of pressure-dependent NO production in capillary stress failure.

### Pressure-dependent mechanism(s) for NOS activation

Activation of NOS by mechanical forces is a hallmark of endothelial mechanotransduction [2–4,28,29] and the signaling pathways that lead to NOS activation include glycocalyx-dependent signaling [3,30], neutral sphingomyelinase (NSMase) [31], IK_ATP_ [32], and TRP channels [33,34]. We have recently demonstrated that inhibition of NSMase prevents pressure-dependent increase in the whole lung filtration coefficient [35]. It is likely therefore, that pressure-dependent activation of NSMase and subsequent release of ceramide is part of the pathway leading to NOS activation.

### Reactive oxygen species in AHF

Little is known about the mechanisms that lead to pulmonary oxidative stress during AHF. We observed that ROS levels were increased in AHF lungs when compared with Sham lungs and we expected that Nox would play a role in ROS production as they are considered the main source for ROS in the vasculature [36]. Our results however indicated that this was not the case: we found no differences in Nox activity or isoforms expression (Nox1 and Nox2) in AHF lungs when compared with Sham lungs. The fact that we did not see increased leukocyte recruitment in BAL or MPO activity in lung tissue and BAL fluid corroborate our findings that Nox 2 is not involved in ROS production during AHF and support the rationale that macrophages are not a source for ROS during AHF.

The evidence clearly suggested that NOS is activated in this model, so we explored eNOS as a potential source for oxidative stress. ROS levels were decreased with pretreatment of AHF rats with L-NAME suggesting eNOS was uncoupled in AHF rat lungs. Immunoblots of lung lysates confirmed a higher eNOS monomer/dimer ratio in this model supporting the idea that uncoupled eNOS was the key element in pulmonary oxidative stress. Treatment of AHF rats with apocynin mitigated lung edema, confirming a role for ROS in barrier failure. We explored the mechanisms that result in eNOS uncoupling, first by evaluating substrate availability (e.g. BH4) and second by assessing caveolae degradation. Supplementation of BH4 to lung lysates reduced ROS formation and confirmed that substrate depletion lead to eNOS uncoupling, thus supporting previous reports in the literature [37].

As eNOS is localized to the caveolae and caveolae dynamics is related to hyperpermeability in lung injury models [38,39], we investigated if caveolae degradation could be part of the mechanism leading to eNOS uncoupling [40]. No changes in caveolin-1 monomer/dimer ratio were found indicating that caveolae degradation is not a determinant for barrier failure in AHF. Our data support previous findings reporting uncoupled eNOS as a mediator of hyperpermeability [7] and as a mechanism in HF [37,41].

## Novelty and clinical significance

**Figure 5:**
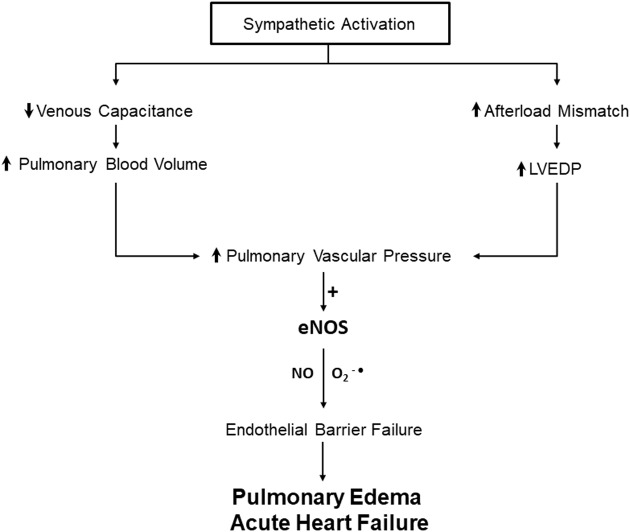
Schematic diagram Schematic diagram illustrating the contribution of mechanotransduction to AHF: elevation of systemic blood pressure by sympathetic activation results in increases in pulmonary vascular pressure due to increased lung blood volume and increased LVEDP. The increase in P_pa_ activates mechanotransduction and leads to excessive eNOS activation that results in NO and superoxide anion (O_2_^−●^) production in the lungs that promote vascular failure culminating in AHF and associated PE.

This is the first report to demonstrate a role for pressure-dependent endothelial hyperpermeability in the pathogenesis of AHF using an intact animal model. The results indicate that pressure-dependent NOS activation contributes to PE development during AHF by increasing NO and ROS production. The observation that L-NAME attenuates PE without altering pulmonary hemodynamics indicate that the increase in pressure *per se* is insufficient to cause lung edema challenging the long-held notion that Starling forces alone can explain rapid and severe PE during AHF. To the contrary, our results suggest that rapid endothelial hyperpermeability, the end result of endothelial mechanotransduction is required in order for the prevailing hydrostatic pressure to cause clinically significant PE (Figure 5). This is a translational study that reveals NOS as a potential target for treating PE during AHF.

## Perspectives

- The endothelial cell is an active participant in AHF.
- Endothelial mechanotransduction contributes to pathogenesis of AHF.
- Targetting endothelial hyperpermeability could be a novel approach for treating AHF.

## Supporting information

**Supplemental Figure 1:** Norepinephrine does not cause hyperpermeability. **A.** To determine the direct effects of norepinephrine (NE) infusion on pulmonary artery pressure, rat lungs were perfused with increasing concentrations of norepinephrine, starting at 10^−8^ mol/L and continuing up to 10^−3^ mol/L. Pulmonary artery pressure did not increase in response to increasing concentration of NE. **B.** To clarify if NE could induce lung edema by non-investigated mechanisms, we perfused rat lungs in situ with a high concentration of NE. Norepinephrine perfusion (NE) did not induce lung edema as evidence by similar wet to dry ratio when compared to control group. A positive control group in which lungs were exposed to pressure and lung edema was developed was also performed. *p<0.05 *vs* C LP. N≥3/group.

**Supplemental Figure 2:** Histological lung injury. Acute Heart Failure rats had increased histological lung injury as evidenced by perivascular cuffing (PVC) and intra-alveolar hemorrhage (IAH). Inhibition of mechanotransduction by pretreatment of hypertensive rats with a bolus of L-NAME (LN+AHF) attenuated both PVC and IAH.

**Supplemental Figure 3:** Table 2. Lung Injury Score in Acute Heart Failure. Lungs have been fixed and stained with Hematoxylin & Eosin for lung injury score. Sections were rated by five blind investigators based on perivascular cuffing and intra-alveolar hemorrhage. Experimental groups: Sham, AHF (acute heart failure) and L-NAME+AHF (L-NAME bolus followed by NE infusion). *p<0.05 *vs* Sham and †p<0.05 *vs* AHF. N=3/group. Abbreviations are as follows: PVC - perivascular cuffing; IAH- intra-alveolar hemorrhage and LIS-lung injury score.

**Supplemental Figure 4:** The Epithelial Barrier is Not Damaged During Acute Heart Failure. Lungs collected from Acute Heart Failure (AHF) rats showed similar **A.** BAL MPO activity; **B.** Lung MPO activity and C. BAL albumin content when compared to sham rats. D. CD45 staining in lung sections of Sham and AHF rats; a negative control was added for comparison. N≥5/group.

**Supplemental Figure 5:** Expression of pro-inflammatory cytokines are not altered during Acute Heart Failure. Pro-inflammatory cytokines were measured in lung lysates of sham and acute heart failure (AHF) lungs. No changes in **A.** IL1β; **B.** MIP2 and **C.** TNFα mRNA were observed in AHF lungs when compared to Sham lungs. N≥4/group.

## Abbreviations

AHF: acute heart failure
Apo: apocynin
BAL: bronchoalveolar lavage
BH4: tetrahydrobiopterin
eNOS: endothelial nitric oxide synthase
EtOH: ethanol
FA: folic acid
HF: heart failure
HR: heart rate
IAH: intra-alveolar hemorrhage
iNOS: inducible nitric oxide synthase
LIS: lung injury score
LVEDP: left ventricular end diastolic pressure
LVESP: left ventricular end systolic pressure
MAP: mean arterial pressure
MIP2: macrophage inflammatory protein 2
mPAP: mean pulmonary artery pressure
MPO: myeloperoxidase
NE: norepinephrine
NOS: nitric oxide synthase
Nox: NADPH oxidase
NSMase: neutral sphingomyelinase
PE: pulmonary edema
P_pa_: pulmonary artery pressure
Ppc: pulmonary capillary pressure
PVC: perivascular cuffing
ROS: reactive oxygen species
TNF-α: tumor necrosis factor-α
W/D: wet to dry

## References

[1] Gandhi S.K., Powers J.C., Nomeir A.M., Fowle K., Kitzman D.W., Rankin K.M. (2001) The pathogenesis of acute pulmonary edema associated with hypertension. N. Engl. J. Med. 344, 17–22 10.1056/NEJM200101043440103 11136955

[2] Dull R.O., Cluff M., Kingston J., Hill D., Chen H., Hoehne S. (2012) Lung heparan sulfates modulate K(fc) during increased vascular pressure: evidence for glycocalyx-mediated mechanotransduction. Am. J. Physiol. Lung Cell. Mol. Physiol. 302, L816–L828 10.1152/ajplung.00080.2011 22160307 PMC3362156

[3] Dull R.O., Mecham I. and McJames S. (2007) Heparan sulfates mediate pressure-induced increase in lung endothelial hydraulic conductivity via nitric oxide/reactive oxygen species. Am. J. Physiol. Lung Cell. Mol. Physiol. 292, L1452–L1458 10.1152/ajplung.00376.2006 17351062

[4] Kim M.H., Harris N.R. and Tarbell J.M. (2005) Regulation of capillary hydraulic conductivity in response to an acute change in shear. Am. J. Physiol. Heart Circ. Physiol. 289, H2126–H2135 10.1152/ajpheart.01270.2004 15994851

[5] Tilton R.G., Chang K.C., LeJeune W.S., Stephan C.C., Brock T.A. and Williamson J.R. (1999) Role for nitric oxide in the hyperpermeability and hemodynamic changes induced by intravenous VEGF. Invest. Ophthalmol. Vis. Sci. 40, 689–696 10067972

[6] Guequen A., Carrasco R., Zamorano P., Rebolledo L., Burboa P., Sarmiento J. (2016) S-nitrosylation regulates VE-cadherin phosphorylation and internalization in microvascular permeability. Am. J. Physiol. Heart Circ. Physiol. 310, H1039–H1044 10.1152/ajpheart.00063.2016 26921435 PMC4867340

[7] Ulker E., Parker W.H., Raj A., Qu Z.C. and May J.M. (2016) Ascorbic acid prevents VEGF-induced increases in endothelial barrier permeability. Mol. Cell. Biochem. 412, 73–79 10.1007/s11010-015-2609-6 26590088 PMC4718828

[8] Stevenson L.W. and Perloff J.K. (1989) The limited reliability of physical signs for estimating hemodynamics in chronic heart failure. JAMA 261, 884–888 10.1001/jama.1989.03420060100040 2913385

[9] Melenovsky V., Andersen M.J., Andress K., Reddy Y.N. and Borlaug B.A. (2015) Lung congestion in chronic heart failure: haemodynamic, clinical, and prognostic implications. Eur. J. Heart Fail. 17, 1161–1171 10.1002/ejhf.417 26467180

[10] Bommakanti N., Isbatan A., Bavishi A., Dharmavaram G., Chignalia A.Z. and Dull R.O. (2017) Hypercapnic acidosis attenuates pressure-dependent increase in whole-lung filtration coefficient (Kf). Pulm Circ. 7, 719–726 10.1177/2045893217724414 28727979 PMC5841912

[11] Chignalia A.Z., Vogel S.M., Reynolds A.B., Mehta D., Dull R.O., Minshall R.D. (2015) p120-catenin expressed in alveolar type II cells is essential for the regulation of lung innate immune response. Am. J. Pathol. 185, 1251–1263 10.1016/j.ajpath.2015.01.022 25773174 PMC4419206

[12] Chignalia A.Z., Oliveira M.A., Debbas V., Dull R.O., Laurindo F.R., Touyz R.M. (2015) Testosterone induces leucocyte migration by NADPH oxidase-driven ROS- and COX2-dependent mechanisms. Clin. Sci. (Lond.) 129, 39–48 10.1042/CS20140548 25700020

[13] Benson M.A., Batchelor H., Chuaiphichai S., Bailey J., Zhu H., Stuehr D.J. (2013) A pivotal role for tryptophan 447 in enzymatic coupling of human endothelial nitric oxide synthase (eNOS): effects on tetrahydrobiopterin-dependent catalysis and eNOS dimerization. J. Biol. Chem. 288, 29836–29845 10.1074/jbc.M113.493023 23965989 PMC3795282

[14] Rassler B., Reissig C., Briest W., Tannapfel A. and Zimmer H.G. (2003) Pulmonary edema and pleural effusion in norepinephrine-stimulated rats–hemodynamic or inflammatory effect? Mol. Cell Biochem. 250, 55–63 10.1023/A:1024942132705 12962143

[15] Rassler B., Reissig C., Briest W., Tannapfel A. and Zimmer H.G. (2003) Catecholamine-induced pulmonary edema and pleural effusion in rats–alpha- and beta-adrenergic effects. Respir. Physiol. Neurobiol. 135, 25–37 10.1016/S1569-9048(03)00062-4 12706063

[16] Viau D.M., Sala-Mercado J.A., Spranger M.D., O’Leary D.S. and Levy P.D. (2015) The pathophysiology of hypertensive acute heart failure. Heart 101, 1861–1867 10.1136/heartjnl-2015-307461 26123135

[17] Kuebler W.M., Uhlig U., Goldmann T., Schael G., Kerem A., Exner K. (2003) Stretch activates nitric oxide production in pulmonary vascular endothelial cells *in situ*. Am. J. Respir. Crit. Care Med. 168, 1391–1398 10.1164/rccm.200304-562OC 12947026

[18] Tarbell J.M., Demaio L. and Zaw M.M. (1999) Effect of pressure on hydraulic conductivity of endothelial monolayers: role of endothelial cleft shear stress. J. Appl. Physiol. (1985) 87, 261–268 10.1152/jappl.1999.87.1.261 10409584

[19] Jiang C., Qian H., Luo S., Lin J., Yu J., Li Y. (2017) Vasopressors induce passive pulmonary hypertension by blood redistribution from systemic to pulmonary circulation. Basic Res. Cardiol. 112, 21 10.1007/s00395-017-0611-8 28258299

[20] Lindsey A.W., Banahan B.F., Cannon R.H. and Guyton A.C. (1957) Pulmonary blood volume of the dog and its changes in acute heart failure. Am. J. Physiol. 190, 45–48 13458406 10.1152/ajplegacy.1957.190.1.45

[21] Krishnamoorthy V., Hiller D.B., Ripper R., Lin B., Vogel S.M., Feinstein D.L. (2012) Epinephrine induces rapid deterioration in pulmonary oxygen exchange in intact, anesthetized rats: a flow and pulmonary capillary pressure-dependent phenomenon. Anesthesiology 117, 745–754 10.1097/ALN.0b013e31826a7da7 22902967 PMC5922446

[22] Cocco G., Jerie P., Amiet P. and Pandolfi S. (2017) Inflammation in heart failure: known knowns and unknown unknowns. Expert Opin. Pharmacother. 18, 1225–1233 10.1080/14656566.2017.1351948 28679294

[23] Dick S.A. and Epelman S. (2016) Chronic heart failure and inflammation: what do we really know? Circ. Res. 119, 159–176 10.1161/CIRCRESAHA.116.308030 27340274

[24] Ichimura H., Parthasarathi K., Issekutz A.C. and Bhattacharya J. (2005) Pressure-induced leukocyte margination in lung postcapillary venules. Am. J. Physiol. Lung Cell. Mol. Physiol. 289, L407–L412 10.1152/ajplung.00048.2005 15879460

[25] Ichimura H., Parthasarathi K., Quadri S., Issekutz A.C. and Bhattacharya J. (2003) Mechano-oxidative coupling by mitochondria induces proinflammatory responses in lung venular capillaries. J. Clin. Invest. 111, 691–699 10.1172/JCI17271 12618523 PMC151903

[26] Jonkam C.C., Bansal K., Traber D.L., Hamahata A., Maybauer M.O., Maybauer D.M. (2009) Pulmonary vascular permeability changes in an ovine model of methicillin-resistant Staphylococcus aureus sepsis. Crit. Care 13, R19 10.1186/cc7720 19222851 PMC2688137

[27] Bhattacharya J. (2003) Pressure-induced capillary stress failure: is it regulated? Am. J. Physiol. Lung Cell Mol. Physiol. 284, L701–2 10.1152/ajplung.00425.2002 12676760

[28] Koo A., Nordsletten D., Umeton R., Yankama B., Ayyadurai S., Garcia-Cardena G. (2013) *In silico* modeling of shear-stress-induced nitric oxide production in endothelial cells through systems biology. Biophys. J. 104, 2295–2306 10.1016/j.bpj.2013.03.052 23708369 PMC3660651

[29] Kim M.H., Harris N.R. and Tarbell J.M. (2005) Regulation of hydraulic conductivity in response to sustained changes in pressure. Am. J. Physiol. Heart Circ. Physiol. 289, H2551–H2558 10.1152/ajpheart.00602.2005 16113077

[30] Yen W., Cai B., Yang J., Zhang L., Zeng M., Tarbell J.M. (2015) Endothelial surface glycocalyx can regulate flow-induced nitric oxide production in microvessels *in vivo*. PLoS ONE 10, e0117133 10.1371/journal.pone.0117133 25575016 PMC4289188

[31] Czarny M. and Schnitzer J.E. (2004) Neutral sphingomyelinase inhibitor scyphostatin prevents and ceramide mimics mechanotransduction in vascular endothelium. Am. J. Physiol. Heart Circ. Physiol. 287, H1344–H1352 10.1152/ajpheart.00222.2004 15142848

[32] Ahn S.J., Fancher I.S., Bian J.T., Zhang C.X., Schwab S., Gaffin R. (2017) Inwardly rectifying K(+) channels are major contributors to flow-induced vasodilatation in resistance arteries. J. Physiol. 595, 2339–2364 10.1113/JP273255 27859264 PMC5374117

[33] Dragovich M.A., Chester D., Fu B.M., Wu C., Xu Y., Goligorsky M.S. (2016) Mechanotransduction of the endothelial glycocalyx mediates nitric oxide production through activation of TRP channels. Am. J. Physiol. Cell Physiol. 311, C846–C853 10.1152/ajpcell.00288.2015 27681180

[34] Yin J., Hoffmann J., Kaestle S.M., Neye N., Wang L., Baeurle J. (2008) Negative-feedback loop attenuates hydrostatic lung edema via a cGMP-dependent regulation of transient receptor potential vanilloid 4. Circ. Res. 102, 966–974 10.1161/CIRCRESAHA.107.168724 18323527

[35] Bommakanti N., Isbatan A., Bavishi A., Dharmavaram G., Chignalia A. and Dull R.O. (2017) Hypercapnic acidosis attenuates pressure-dependent increase in whole-lung filtration coefficient (Kf). Pulm Circ., 7, 719–726 10.1177/2045893217724414 28727979 PMC5841912

[36] Li H., Horke S. and Forstermann U. (2014) Vascular oxidative stress, nitric oxide and atherosclerosis. Atherosclerosis 237, 208–219 10.1016/j.atherosclerosis.2014.09.001 25244505

[37] Yamamoto E., Hirata Y., Tokitsu T., Kusaka H., Sakamoto K., Yamamuro M. (2015) The pivotal role of eNOS uncoupling in vascular endothelial dysfunction in patients with heart failure with preserved ejection fraction. Int. J. Cardiol. 190, 335–337 10.1016/j.ijcard.2015.04.162 25935623

[38] Maniatis N.A., Kardara M., Hecimovich D., Letsiou E., Castellon M., Roussos C. (2012) Role of caveolin-1 expression in the pathogenesis of pulmonary edema in ventilator-induced lung injury. Pulm. Circ. 2, 452–460 10.4103/2045-8932.105033 23372929 PMC3555415

[39] Maniatis N.A., Chernaya O., Shinin V. and Minshall R.D. (2012) Caveolins and lung function. Adv. Exp. Med. Biol. 729, 157–179 10.1007/978-1-4614-1222-9_11 22411320 PMC3449096

[40] Cassuto J., Dou H., Czikora I., Szabo A., Patel V.S., Kamath V. (2014) Peroxynitrite disrupts endothelial caveolae leading to eNOS uncoupling and diminished flow-mediated dilation in coronary arterioles of diabetic patients. Diabetes 63, 1381–1393 10.2337/db13-0577 24353182 PMC3964507

[41] Yamamoto E., Kataoka K., Shintaku H., Yamashita T., Tokutomi Y., Dong Y.F. (2007) Novel mechanism and role of angiotensin II induced vascular endothelial injury in hypertensive diastolic heart failure. Arterioscler. Thromb. Vasc. Biol. 27, 2569–2575 10.1161/ATVBAHA.107.153692 17932313

