# Supplementary material for: Pressure-dependent NOS activation contributes to endothelial hyperpermeability in a model of acute heart failure
Source: Biosci Rep. 2018 Nov 23;38(6):BSR20181239. doi: 10.1042/BSR20181239 (PMC6250809; doi:10.1042/BSR20181239)

SUPPLEMENTAL MATERIAL

Pressure-dependent NOS activation contributes to endothelial hyperpermeability in a model of acute heart failure

Andreia Zago Chignalia, PhD[1]; Ayman Isbatan[1]; Milan Patel[1]; Richard Ripper[1,2], Jordan Sharlin MD[1], Joelle Shosfy MD[1], Barry A. Borlaug MD[3], Randal O Dull MD, PhD[1,4].

1. Department of Anesthesiology, University of Illinois at Chicago, Chicago, IL, 60612
2. Jesse Brown Veterans Affair Medical Center, 820 S Damen Ave., Chicago, IL 60612.
3. Mayo Clinic and Foundation, 200 First St SW, Rochester, MN 55905 .
4. Department of Anesthesiology, University of Arizona COM and Banner-University Medical Center. Tucson, AZ 85724.

Running title: eNOS during acute heart failure

Corresponding author: Andreia Zago Chignalia. Department of Anesthesiology, University of Illinois at Chicago. 1740 West Taylor Street, Suite 3200. Chicago, Il, 60612. Telephone 312-996-4020. Email: andreiac@uic.edu

Expanded Methods Section

Reagents

Chemicals were the highest grade available. L-N^G-Nitroarginine methyl ester (L-NAME) and lucigenin were bought from Cayman Biochemicals (Ann Arbor, MI); (±)-Norepinephrine (+)-bitartrate salt, protease inhibitor cocktail, Dihydroethidium (DHE), Krebs-ringer bicarbonate buffer were purchased from Sigma Co, Ltd (St Louis, MO); Apocynin was bought from Calbiochem; bovine serum albumin was from Proliant Biologicals (Boone, IA); anti-nitrotyrosine, anti-iNOS and anti phospho eNOS antibodies were from EDM Millipore (Billerica, MA); β-actin, anti eNOS antibodies were purchased from Cell Signaling (Danvers, MA); Albumin fluorescent kit was from Active Motif (Carlsbad, CA); *o*-dianisidine dihydrochloride was from TCI America (Portland, OR), hydrogen peroxide (H_2O_2) and hexadecyltrimethylammonium bromide (HTBA) were purchased from Fisher Scientific (Waltham, MA)

Animals

All animal studies were approved by the University of Illinois Institutional Animal Care and Use Committee. Male Sprague Dawley male rats weighing 250-300 g were used. Rats were divided in the following groups: Sham; Acute Heart Failure (AHF); L-NAME + AHF (L-NAME + AHF); L-NAME; Ethanol; Apocynin (Apo); Apocynin + AHF (Apo + AHF); Folic Acid and Folic Acid + AHF.

Acute Heart Failure Model

Rats were anesthetized with isoflurane, a tracheotomy was performed and they were mechanically ventilated with room air at RR = 60/min, PIP = 10 cm H_2O, PEEP = 3 cm H_2O. A carotid artery and jugular vein catheter was inserted for recording of blood pressure and drug administration, respectively. Rats received a norepinephrine infusion starting at 7 μg/kg/min and titrated to maintain a MAP of 150 mmHg for a period of 2 hours. Shams were submitted to same anesthesia and surgical procedures but received an infusion of lactate ringers instead of norepinephrine. L-NAME was administered as a bolus targeting a plasma concentration of 200 µmol/L before norepinephrine infusion (Supplemental Figure 1)

Rat In-Situ Perfused Lung Preparation

Isolated perfused lung preparation was performed as previously described(1) with minor modifications. Briefly, animals were anesthetized with ketamine-xylazine (90:10 mg/kg) and mechanically ventilated. The chest and pericardial sac were opened and ligatures were placed around the aorta and pulmonary artery. Animals were heparinized (heparin 200U) and exsanguinated. The left atrium and the pulmonary artery were cannulated and lungs were perfused with Krebs-Ringer-bicarbonate solution supplemented with 3% bovine serum albumin. Pulmonary artery (P_{PA}) and left atrial pressures (P_{LA}) were measured continuously via in-line pressure transducers (P-75, Harvard Apparatus, Natick, MA) connected to an analog-to-digital board. Left atrial

pressure was set to 3 cm of water pressure for the entire experimental protocol. An in-line ultrasonic flow probe (Transonic, Ithaca, NY) was placed in the pulmonary artery cannula and vascular pressures data was recorded in real time using a custom-written program (LabVIEW, National Instruments, Austin, TX).

Aliquots of NE were serially added to the perfusate reservoir to produce a dose range from 10^{-8} mol/L to 10^{-3} mol/L; the concentration of NE was increased every 10 minutes and PA pressure was recorded as described above. Thus, the direct effects of NE on rat pulmonary vasculature pressure were determined.

Arterial Blood Gas Analysis

Whole blood was collected immediately after animal was anesthetized and every 30 minutes after starting specific treatment (Norepinephrine or L-NAME) or lactated ringers infusion. Blood gases, hematocrit (HCT) levels and pH were measured using a GEM Premier 3000 (Instrumentation Laboratory, Orangeburg, NY) according to manufacturer's instructions.

Wet-to-dry ratio

Lungs and bowel were collected at the end of the experiment and wet weights were obtained immediately after organ collection. Samples were placed in an oven at 60°C for 24 hours. Lung and bowel dry weights (LDW and BDW, respectively) were measured and the wet-to-dry (W/D) ratio was determined

Plasma Volume Determination

During the animal surgery, a constant infusion of fluid (lactate ringers) and saline boluses were given to the rats to maintain MAP and HCT. The amount of fluid required for each animal differed requiring the changes in plasma volume to be corrected for the individual infusion and boluses. We developed a series of calculations to estimate the amount of total plasma loss that occurs during the hypertensive episode and this served as an indicator of systemic fluid escape to the interstitial space. Total blood volume (TBV) was calculated prior to surgery (equation 1).

$$TBV=70mL/Kg \qquad (1)$$

The plasma volume was derived every 30 minutes based on HCT levels taken from each blood gas. The TBV was corrected for the volume of blood (cTBV) used in each ABG measurement (0.25 mL), by equation 2

$$cTBV=TBV-0.25 \qquad (2)$$

The estimated plasma volume (ePV) was calculated by equation 3

$$ePV= (1-(HCT/100))*cTBV \qquad (3)$$

and was then corrected (cPV) for the amount of fluid that was given during that 30 minute period by adding the volume of infused drugs (VDI; norepinephrine and/or L-NAME), fluid (VFI, lactate ringers) and saline boluses (VSI) as specified in equation 4

$$cPV = ePV + VDI + VFI + VSI \qquad (4)$$

The change in plasma volume (ΔPV) was derived by subtracting the ePV from the cPV (Equation 5) yielding ΔPV.

$$\Delta PV = cPV - ePV \quad (5)$$

The ΔPV from baseline to 120 minutes is presented for all groups.

Lung Injury Score

Lungs were isolated and instilled with10% formalin under a pressure gradient of 30 cm H_2O for 10 minutes via the trachea. Lungs were then stored in 10% formalin buffer at room temperature for 24 hours and transferred to 70% ethanol, 4^oC, until processed by UIC Histology Core. Lung sections were stained with hematoxylin eosin and imaged using an upright microscope (OLYMPUS BX51). Lung injury was assessed in the ventral and dorsal aspects of the lung by five blinded investigators based on a modification of the American Thoracic Society (ATS) scoring(2) using perivascular cuffing (PVC) and intra-alveolar hemorrhage (IAH) as primary variables. Total lung injury score (LIS) was determined by the weighted average of PVC and IAH, where LIS= [(PVCx20) + (IAHx14)]/68.

Bronchoalveolar lavage (BAL) albumin content

BAL albumin content was assessed using a fluorimetric detection kit (Active Motif, Carlsbad CA) according to manufacturer's instructions.

Myeloperoxidase (MPO) activity

MPO activity was measured in lung tissue and in BAL. Samples were collected and immediately frozen until processing. Lungs were homogenized in 5% HTBA buffer, sonicated three times for 15 seconds on ice and centrifuged at 14,000 x g for 10 minutes at 4°C. The pellet was re-suspended in HTBA buffer, subjected to two cycles of 20 minutes of freezing/thawing and separated by centrifugation. The MPO activity was measured in the supernatant of the third cycle using a 96-well plate. Homogenized lung tissue or BAL samples (200 uL) were added to each well. *o*-Dianisidine dihydrochloride with 0.0005% hydrogen peroxide in phosphate buffer (100 uL) was then added to each sample. Absorbance change was measured at 460 nm for 3 minutes. MPO activity was expressed as O.D./mg tissue/mL of buffer or in absolute O.D. values (BAL).

Assessment of mechanotransduction activation

NOS activation is a central component of endothelial mechanotransduction, therefore, NOS expression and activity was assessed by immunoblot. Indirect measurement of nitric oxide production via the quantification of nitration of tyrosine residues was also assessed. Lungs were homogenized in RIPA buffer supplemented with proteases inhibitor cocktail, PMSF, sodium orthovanadate and sodium fluoride. Samples were centrifuged for 15 min, 13000 rpm at 4°C. Protein was measured by Bradford assay and samples

were prepared with Biorad Laemmli buffer + β-mercaptoethanol. Samples were separated in an acrylamide gel and transferred to nitrocellulose membranes. Membranes were incubated with primary antibody at 4°C overnight, washed and incubated with secondary antibody for 1h at room temperature. Signal was detected by chemiluminescence using the LI-COR system. Band intensities were quantified using Image Studio software (LI-COR).

eNOS uncoupling

To assess whether eNOS is uncoupled during pressure-induced acute heart failure we performed a low temperature gradient gel following standard western blot techniques as previously described(3). Samples were prepared using laemni buffer and b-mercaptoethanol but not boiled and the detection of eNOS monomer/dimer was performed. Protein lysates were resolved using a 4-20% Tris-glycine gradient gel (Biorad). All gels and buffers were pre-equilibrated to 4 °C before electrophoresis, and the buffer tank was placed in an ice bath during electrophoresis and transfer to maintain the gel temperature below 15 °C. Membranes were incubated with anti eNOS antibody (cell signaling). Signal was detected by chemiluminescence using LiCor system. Band intensities were measured using Image Studio software (LiCor).

Lucigenin enhanced chemiluminescence

Lung tissue was collected and immediately frozen until analysis. In the day of the experiment, lungs were homogenized in lysis buffer (20 mmol/L of KH2PO4, 1 mmol/L of EGTA, 1 μg/mL of aprotinin, 1 μg/mL of leupeptin, 1 μg/mL of pepstatin, and 1 mmol/L of PMSF), incubated in ice for 30 min and centrifuged for 15 min, 13000rpm at 4C. Supernatant was collected and used for analysis. Fifty microliters of the sample were added to a suspension containing 175 μL of assay buffer (50 mmol/L of KH2PO4, 1 mmol/L of EGTA, and 150 mmol/L of sucrose) and lucigenin (5 μmol/L). NADPH (10−4 mol/L) was added to the suspension (300 μL) containing lucigenin. Luminescence was measured every 18 seconds for 3 minutes by a luminometer (SpectraMax M5, Molecular Devices) before and after stimulation with NADPH. A buffer blank was subtracted from each reading. The results are expressed as counts per milligram of protein (percentage of control). To determine NOS-dependent superoxide generation, rats were treated with L-name and lucigenin enhanced chemiluminescence was performed.

NADPH oxidase activity

Membrane fractions were isolated from frozen lungs using the Mem-Per Plus kit (Thermo Scientific). NADPH oxidase activity was assessed by the kinetics of DHE oxidation in a plate fluorometer (GENios Pro, Tecan). 150μL of the membrane extract were added to each well, along with 0.12 μL of DHE (10 mM), 25 μg/mL of DNA and 101.88 μL of phosphate buffer (50 mM, pH 7.4). Fluorescence was read for 30 minutes (λexc.485 nm, λem.595 nm). Three μL of NADPH 2x10-3 mol/L were then added and a new reading was performed after

30 min. The delta for the fluorescence was calculated (∆RFU =RFU after NADPH – RFU before NADPH). ∆RFU was normalized by protein content in the sample.

Statistical Analysis

Data are presented as mean ± SD. Groups were compared using one-way ANOVA and comparison of group means was done using Tukey post-test or t-test. $P<0.05$ was considered statistically significant.

Legends to Figures

Supplemental Figure 1. Norepinephrine does not cause hyperpermeability*.* A. To determine the direct effects of norepinephrine (NE) infusion on pulmonary artery pressure, rat lungs were perfused with increasing concentrations of norepinephrine, starting at 10^{-8} mol/L and continuing up to 10^{-3} mol/L. Pulmonary artery pressure did not increase in response to increasing concentration of NE. **B.** To clarify if NE could induce lung edema by non-investigated mechanisms, we perfused rat lungs in situ with a high concentration of NE. Norepinephrine perfusion (NE) did not induce lung edema as evidence by similar wet to dry ratio when compared to control group. A positive control group in which lungs were exposed to pressure and lung edema was developed was also performed. *$p<0.05$ *vs* C LP. N≥3/group.

Supplemental Figure 2. Histological lung injury. Acute Heart Failure rats had increased histological lung injury as evidenced by perivascular cuffing (PVC) and intra-alveolar hemorrhage (IAH). Inhibition of mechanotransduction by pretreatment of hypertensive rats with a bolus of L-NAME (LN+AHF) attenuated both PVC and IAH.

Supplemental Figure 3. Table 2. Lung Injury Score in Acute Heart Failure: Lungs have been fixed and stained with Hematoxylin & Eosin for lung injury score. Sections were rated by five blind investigators based on perivascular cuffing and intra-alveolar hemorrhage. Experimental groups: Sham, AHF (acute heart failure) and L-NAME+AHF (L-NAME bolus followed by NE infusion). *$p<0.05$ *vs* Sham and †$p<0.05$ *vs* AHF. N=3/group. Abbreviations are as follows: PVC – perivascular cuffing; IAH- intra-alveolar hemorrhage and LIS-lung injury score.

Supplemental Figure 4. The Epithelial Barrier is Not Damaged During Acute Heart Failure. Lungs collected from Acute Heart Failure (AHF) rats showed similar **A.** BAL MPO activity; **B.** Lung MPO activity and **C.** BAL albumin content when compared to sham rats. D. CD45 staining in lung sections of Sham and AHF rats; a negative control was added for comparison. N≥5/group.

Supplemental Figure 5. Expression of pro-inflammatory cytokines are not altered during Acute Heart Failure. Pro-inflammatory cytokines were measured in lung lysates of sham and acute heart failure (AHF) lungs. No changes in **A.**

IL1β; **B.** MIP2 and **C.** TNFα mRNA were observed in AHF lungs when compared to Sham lungs. N≥4/group.

References

1. Dull RO, Cluff M, Kingston J, Hill D, Chen H, Hoehne S, et al. Lung heparan sulfates modulate K(fc) during increased vascular pressure: evidence for glycocalyx-mediated mechanotransduction. Am J Physiol Lung Cell Mol Physiol. 2012;302(9):L816-28.

2. Matute-Bello G, Downey G, Moore BB, Groshong SD, Matthay MA, Slutsky AS, et al. An official American Thoracic Society workshop report: features and measurements of experimental acute lung injury in animals. Am J Respir Cell Mol Biol. 2011;44(5):725-38.

3. Benson MA, Batchelor H, Chuaiphichai S, Bailey J, Zhu H, Stuehr DJ, et al. A pivotal role for tryptophan 447 in enzymatic coupling of human endothelial nitric oxide synthase (eNOS): effects on tetrahydrobiopterin-dependent catalysis and eNOS dimerization. J Biol Chem. 2013;288(41):29836-45.

Supplemental Figure 1

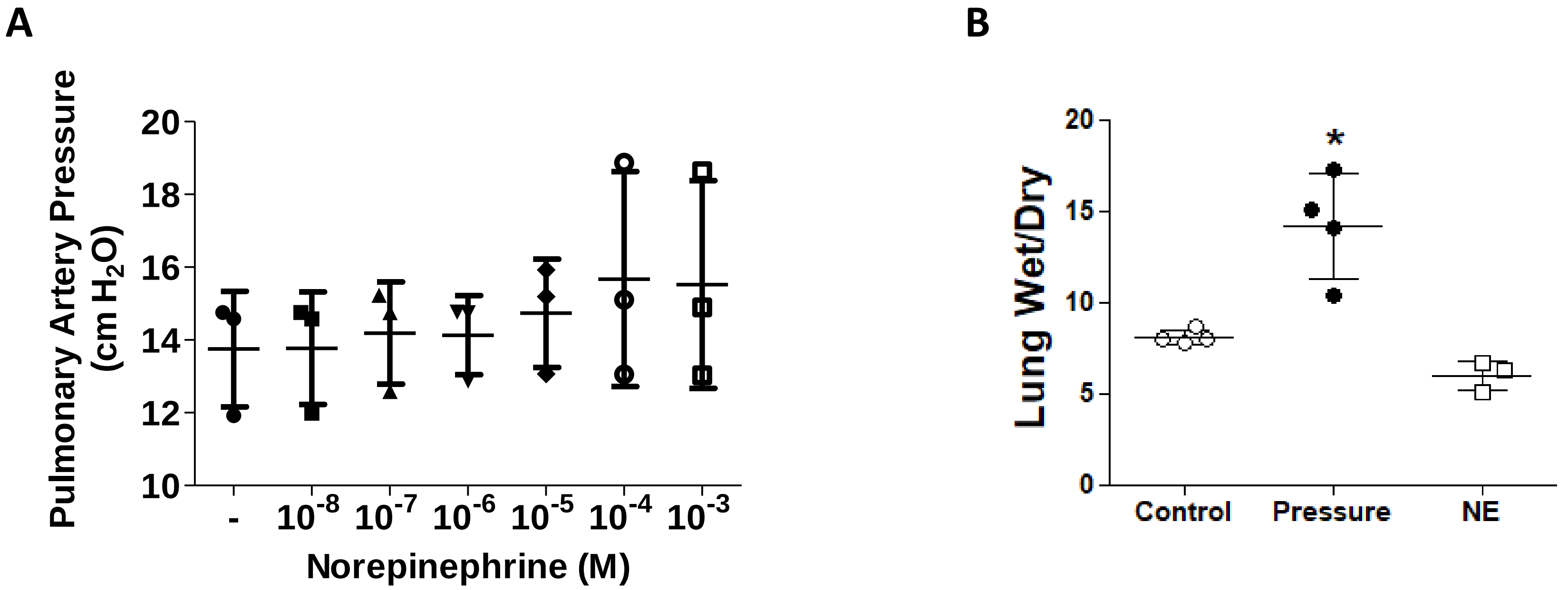

Supplemental Figure 2

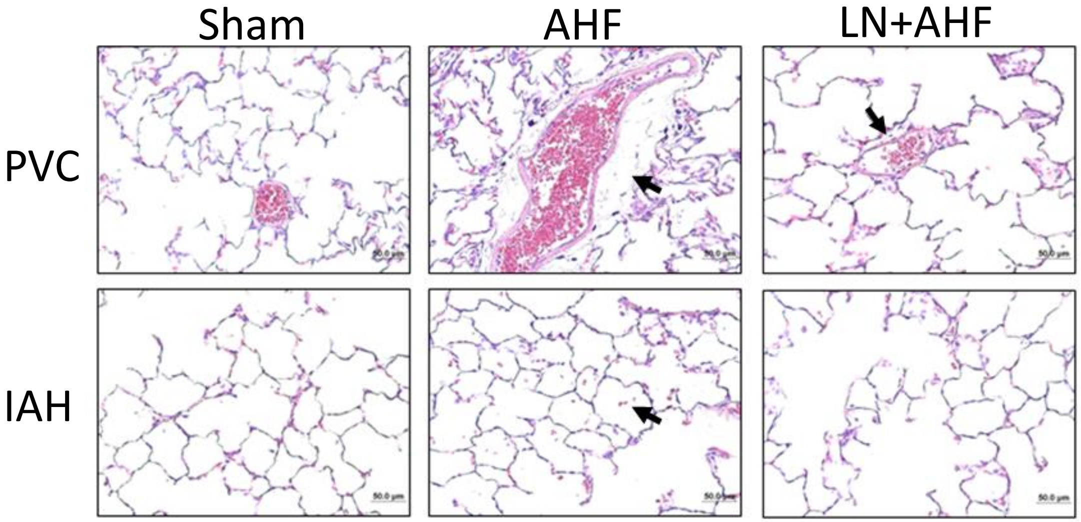

Table 2. Lung Injury Score in Acute Heart Failure

	SHAM	NE	LNAME+NE
**Dorsal PVC**	0.448±0.2	0.873±0.1*	0.789±0.1*
**Dorsal IAH**	0.199±0.1	0.603±0.2*	0.291±0.2*†
**Dorsal LIS**	0.172±0.1	0.381±0.1*	0.287±0.1*
**Ventral PVC**	0.556±0.2	0.782±0.7	0.642±0.1
**Ventral IAH**	0.275±0.1	0.320±0.2	0.430±0.1
**Ventral LIS**	0.219±0.1	0.291±0.1	0.272±0.1

Supplemental Figure 4

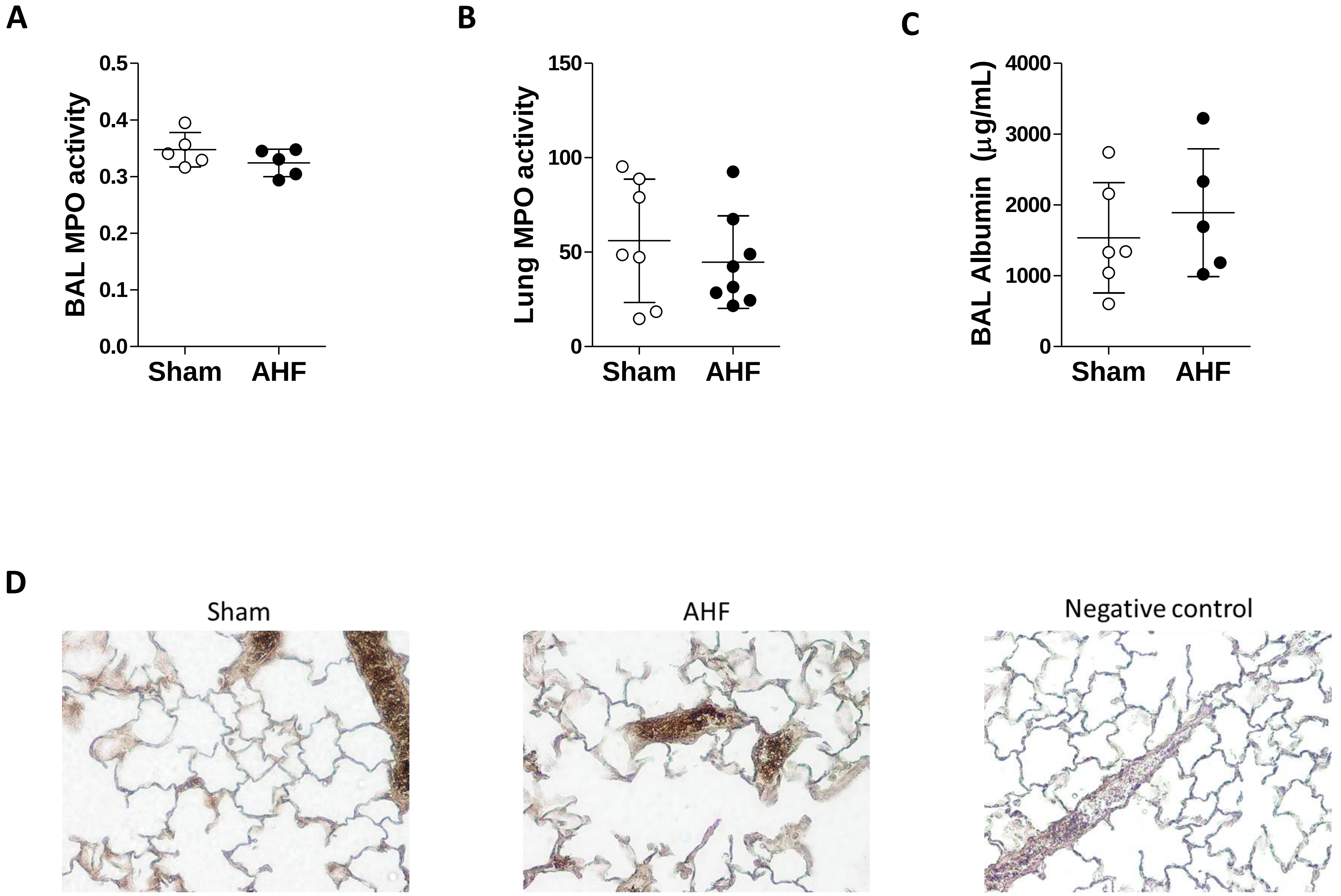

Supplemental Figure 5

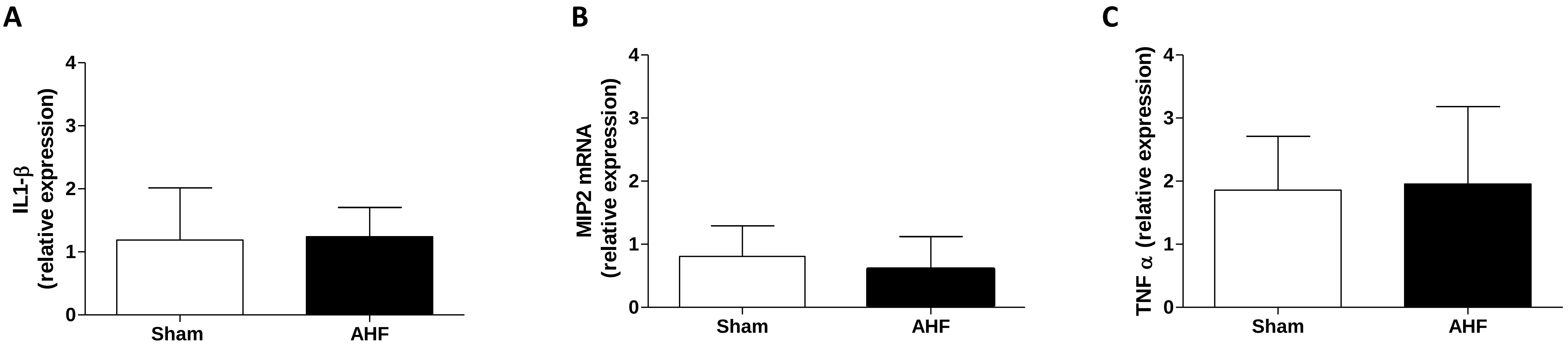

Supplement: Supplementary file 1 [file bsr20181239_Supp1.pdf]
